# Cellular State Transformations Using Deep Learning for Precision Medicine Applications

**DOI:** 10.1016/j.patter.2020.100087

**Published:** 2020-08-17

**Authors:** Colin Targonski, M. Reed Bender, Benjamin T. Shealy, Benafsh Husain, Bill Paseman, Melissa C. Smith, F. Alex Feltus

**Affiliations:** 1 Department of Electrical and Computer Engineering, Clemson University, Clemson, SC 29634, USA; 2 Department of Biomedical Data Science and Informatics, Clemson University, Clemson, SC 29634, USA; 3 Paseman & Associates, Saratoga, CA 95070, USA; 4 Department of Genetics and Biochemistry, Clemson University, Clemson, SC 29634, USA; 5 Center for Human Genetics, Clemson University, Greenwood, SC 29646, USA

**Keywords:** deep learning, generative adversarial networks, tumor biology, gene expression, precision medicine, renal cell carcinoma

## Abstract

We introduce the Transcriptome State Perturbation Generator (TSPG) as a novel deep-learning method to identify changes in genomic expression that occur between tissue states using generative adversarial networks. TSPG learns the transcriptome perturbations from RNA-sequencing data required to shift from a source to a target class. We apply TSPG as an effective method of detecting biologically relevant alternate expression patterns between normal and tumor human tissue samples. We demonstrate that the application of TSPG to expression data obtained from a biopsy sample of a patient's kidney cancer can identify patient-specific differentially expressed genes between their individual tumor sample and a target class of healthy kidney gene expression. By utilizing TSPG in a precision medicine application in which the patient sample is not replicated (i.e., n=1), we present a novel technique of determining significant transcriptional aberrations that can be used to help identify potential targeted therapies.

## Highlights

• We present the Transcriptome State Perturbation Generator (TSPG) application
• We apply TSPG to The Cancer Genome Atlas data to perturb gene expression states
• TSPG was used to learn patient-specific (*n* = 1) gene expression tumor alterations

## The Bigger Picture

Deep learning has shown tremendous success in image and natural language processing; however, attempts to apply the tools of machine learning to better understanding biological systems are still in the stage of early adoption. We propose a novel deep-learning tool that can be used to process samples of RNA-sequencing data. By applying the Transcriptome State Perturbation Generator to human samples, we show that deep learning derives insight into the gene expression shifts required for transition between two biological conditions (e.g., normal versus tumor). RNA-sequencing data derived from a single patient's tumor were analyzed using this tool to determine gene expression aberrations specific to that patient's tumor. As medicine shifts from cohort-based population studies to individual-based precision treatments, our example demonstrates that deep learning is a powerful ally in the quest to understand how complex biological systems have shifted for a single patient.

## Graphical Abstract

**Figure:**
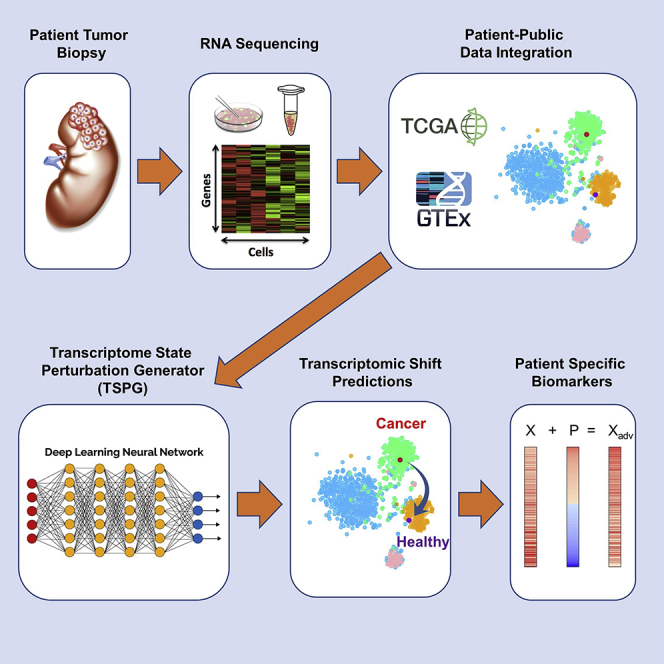

## Teaser

Cells express genes in unique combinations that enable distinct functions. Using machine learning, we present an algorithm that takes a source gene expression snapshot and learns how to change it to mimic a target snapshot. We applied the Transcriptome State Perturbation Generator algorithm to learn which genes have changed in a single patient's tumor relative to a normal tissue sample. By knowing which gene expression changes are required to leave a normal state in a single person, it is possible to design therapeutic strategies tailored for that patient.

## Introduction

Nearly 4 years prior to the completion of the Human Genome Project,^1^ Francis Collins published an article that defined the potential for a new field of precision medicine, titled “Medical and societal consequences of the Human Genome Project.” Dr. Collins made the preemptive observation that “The transition from genetics to genomics marks the evolution from an understanding of single genes and their individual functions to an understanding of the actions of multiple genes and their control of biologic systems.”^2^ Although significant attempts have been made to achieve this transition, the vast majority of cancer research is often centered on the traditional reductionist approach of looking for focused genetic mutations (e.g., tumor suppressors or oncogenes) that are highly correlated with the heredity or progression of certain cancers.^3^ Identification of these cancer-specific genetic aberrations has resulted in a revolution in our understanding of cancer, although these revelations only tell a part of the story. Every tumor presents complex genomic changes that affect multiple genes through mutation as well as alterations in their expression patterns, resulting in unique genomic profiles of each individual patient's cancer. It is insufficient, therefore, to look for single genetic alterations that can be linked to a cancer's progression. Rather, cancers are the result of complex and chaotic changes in the genomic and epigenetic homeostasis of cells that resemble Rube-Goldberg devices with many intricately interconnected components.^3^, ^4^, ^5^

Seeking to provide context to these genomic interactions, there have been several initiatives in recent years to characterize the genomes of different types of human cancers. The National Institutes of Health pioneered the creation of The Cancer Genome Atlas (TCGA) in 2005 in an effort to provide the molecular and physical map of cancer aberrations to improve our ability to diagnose, treat, and prevent cancer.^4^^,^^6^ TCGA and other data repositories including the Cancer Cell Line Encyclopedia and the International Cancer Genome Consortium (ICGC) have collected multidimensional data on thousands of samples of different cancers.^7^^,^^8^ By grouping cancers by their relevant genomic mutations, these resources have been used to identify new mutations present in different tumors and are beginning to characterize the different cellular pathways in which they appear.^9^ Furthermore, the quantity of data that is now available allows researchers to look at statistical averages within the different groups of tumors, identifying significant aberrations that are characteristic of a tumor subtype across many samples. The flood of information that has resulted from these studies has led to insights that are currently driving advances in therapeutic strategies and diagnostic tools; however, the practical application of this enormous volume of genomic information remains largely unrealized.

Precision medicine promises to change the course of cancer care by allowing doctors to select treatments that are most likely to help patients based on a genetic understanding of their disease.^10^ This reflects a fundamental change in focus from the current standard of medical practice, which largely relies on cohort-based epidemiological studies in which the genetic variability of individuals is largely ignored, resulting in population-based conclusions.^4^ As more knowledge is gathered about how genetics affects cancer progression, it strengthens the conclusion that each individual tumor has its own set of unique mutations, and furthermore that it is this variation that makes similar types of cancer respond differently to the same treatments. Coupled with the fact that humans differ from one another by an average of 6 million nucleotides in the genome, prior to the cascade of unique changes that occur in the cancer transcriptome, it becomes clear that each person must be treated as a unique individual and not as a statistical average if we are to begin to understand the oddities of cancer on a case-by-case basis.^11^

One of the most challenging barriers in analyzing genomic data is the remarkably low signal-to-noise ratio (SNR). Tumors each harbor a combination of cancer-causing, or driver, mutations and largely irrelevant passenger mutations that do not contribute to the oncogenic potential of the cells. Distinguishing one from the other is critically important, but it is nearly impossible to accomplish when looking at a single patient sample in isolation. A precision medicine approach to cancer genomics is therefore plagued with the fact that a single patient sample is not replicated (i.e., n=1).^12^ There is a need, therefore, for a method of examining the entire genome of a patient-specific tumor that leverages these large collections of genomic data to mitigate the low SNR.

RNA sequencing (RNA-seq) provides a useful insight into the gene expression state of a biological sample by measuring tens of thousands of unique RNA transcripts.^13^ In cancer, different tumors may have relatively similar protein coding sequence (CDS) profiles, but the expression patterns of those genes may be significantly different across tumors. This can result in an even lower SNR in expression data than is seen in exome CDS data while simultaneously increasing the variability between samples.^14^ Traditional methods of high-dimensional analysis are challenging to use on RNA-seq datasets on account of this, as well as the high feature-to-sample ratio apparent in biological systems. Modern machine-learning and deep-learning approaches have begun to surmount these obstacles and are increasingly used to cluster and classify gene expression profiles into meaningful groups that correspond with metadata labels such as tissue source or particular phenotype.^15^, ^16^, ^17^

Deep learning^18^ has had tremendous success in image processing^19^^,^^20^ and natural language processing^21^^,^^22^ tasks due to its ability to abstract high level features from high-dimensional and noisy datasets. Deep learning has also been used to develop powerful generative models such as variational autoencoders (VAEs)^23^ and generative adversarial networks (GANs).^24^ GANs, a focus of this work, consist of a generator that captures a data distribution and a discriminator that estimates the probability that a sample came from the training data rather than the generator. As training progresses, the generator produces increasingly realistic data while the discriminator becomes more adept at distinguishing real from fake. GANs have surged in popularity in the field of computer vision due to several works that can create exceptionally realistic images.^25^, ^26^, ^27^

While deep-learning models are being successfully applied in a variety of fields, surprising vulnerabilities exist within trained models. Adversarial samples (or adversarial examples) can be designed to purposefully cause the deep-learning model to incorrectly classify the sample.^28^^,^^29^ An adversarial sample consists of a real sample, whose class is the source class, with an added perturbation that causes the model to classify the perturbed sample as a different class, which is the target class. In other words, adversarial samples are used to “trick” a neural network into confidently choosing a target class. For example, an image *x*, classified as a horse by a neural network, can be subtly perturbed into $x_{\text{adv}}=x+p$ that is classified by the same neural network as a human. Previous works^29^, ^30^, ^31^ have led to Xiao et al.^32^ proposing AdvGAN, an adversarial sample generation framework, to produce perceptually realistic images in an efficient manner using GANs.

Within the field of molecular biology a limited amount of work involving generative models has been reported. Ghahramani et al.^33^ proposed a Wasserstein GAN^34^ to integrate epidermal datasets by generating samples that cover the full diversity of cell types. Additionally, the authors used the generative model for both dimensionality reduction and to observe the effect of cell state perturbations on gene expression. Ghasedi Dizaji et al.^35^ introduced a semi-supervised approach to generate gene expression profiles of target genes using landmark genes and GANs.

In this report, we contribute to this emerging field by adapting the AdvGAN framework to model the transition between source and target tissue transcriptome states. Furthermore, we demonstrate how a single patient sample can be perturbed to resemble the expression state of a larger cohort by leveraging TCGA data as the training set for the model. By using TCGA data to train our network, we show that a model can be trained to recognize biologically relevant patterns in RNA-seq data and can effectively perturb a patient's tumor RNA-seq sample to resemble the expression patterns of normal tissue. Those perturbations can then be analyzed to determine significant transcriptional changes that occur during tumor progression, thus solving the n=1 precision medicine issue.

## Results

### Validation of TSPG

We examined the raw adversarial generation capabilities using 50 Hallmark gene sets defined in the Broad Institute Molecular Signatures Database (MSigDB).^36^ First, we extracted the RNA-seq expression profiles for Hallmark gene subsets from the normal human tissue Genotype-Tissue Expression (GTEx) repository.^37^ Table 1 contains the results for various Hallmark gene subsets expression profiles as feature input to the TSPG framework and corresponding target sample class. The furthest right column, *f* Accuracy, represents the target model accuracy when the input is perturbed from any input source (i.e., all samples from the dataset) to the labeled target class. Thus the accuracy represents the ability of the generator to “trick” the target model into classifying a sample as a specified class.

**Table 1:** Accuracy of Target Network on Perturbed GTEx Datasets

Gene Set	Genes	Target Class	*f* Accuracy (%)
Hallmark Hedgehog Signaling	36	nerve-tibial	100
Hallmark Peroxisome	107	brain-spinal cord	100
Hallmark Apoptosis	161	lung	99.9
Hallmark E2F Targets	200	artery-coronary	99.8
Hallmark All	4386	thyroid	100
Hallmark All	4386	heart-left ventricle	100

The high classification accuracy suggested that the generator was able to exploit the decision boundary between any two classes in the target model. While this was in itself interesting, we were led to three new questions concerning the nature of these perturbations: (1) Do the perturbed samples follow the distribution of the target class? (2) Are these perturbations nonsensically changing expression values to satisfy the criterion of tricking a target model? (3) Do the most highly perturbed genes encode biological function of the target condition?

**Figure 1:**
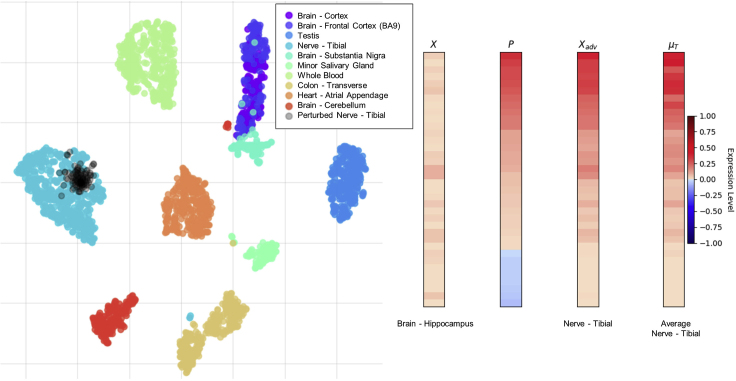
Adversarial Generation for Nerve-Tibial Target Using the Hallmark Hedgehog Signaling Gene Set t-SNE plot of original and perturbed samples using the Hallmark Hedgehog Signaling gene set (left). Heatmap of cellular transformation from Brain-Hippocampus to Nerve-Tibial (right). Perturbation (*P*) ranges from [−1,1], which is added to original sample (*X*), then adversarial sample ($X_{\text{adv}}$) is clipped to [0,1]. The mean expression vector ($\mu_{T}$) of the target class (Nerve-Tibial) is shown.

To test whether the adversarial samples exhibited high-dimensional structure similar to that of the original target samples, we used the dimensionality reduction and visualization tool t-SNE (t-distributed stochastic neighbor embedding).^38^ t-SNE allowed us to determine whether perturbed samples clustered with the distribution of the target condition. In Figure 1, ten different classes from the GTEx dataset were plotted as well as 100 randomly chosen adversarial samples that were created from varying source tissues in the original dataset. Each sample was originally 36 dimensions representing the 36 genes in the Hallmark Hedgehog signaling gene set. We observed that the original (unperturbed) Nerve-Tibial samples (dark cyan) and the adversarial (perturbed) Nerve-Tibial samples (black) formed a cluster on the left side of the plot. The adversarial (black) points were originally 100 different samples from the GTEx dataset of varying class description. These were passed through the generative process, transforming these points to the target condition. It is evident from Figure 1 that the perturbed samples follow a similar data distribution as the original target condition.

The right side of Figure 1 shows the step-by-step process of generating an adversarial sample. In this case, *x* is a heatmap of gene expression values from an original sample from the GTEx dataset of class type Brain-Hippocampus. *x* was passed through the generator to produce *P*, which was then added to *x* to create adversarial sample $x_{\text{adv}}$. *P* ranges between [−1,1] to allow the generator to entirely “activate” or “silence” any gene. $x_{\text{adv}}$ was clipped to valid expression values in the range of [0,1]. This generated sample $x_{\text{adv}}$ was then classified as target class *t* by a pretrained model (the target model). Perturbation values *P* describing the exact gene expression changes remained, on a per-gene basis, which occurred in order to transform Brain-Hippocampus tissue to Nerve-Tibial tissue. Additionally we show $\mu_{T}$, the mean expression vector for the target class (Nerve-Tibial in this example), for direct visual comparison between an adversarial sample $x_{\text{adv}}$ and the target class.

**Figure 2:**
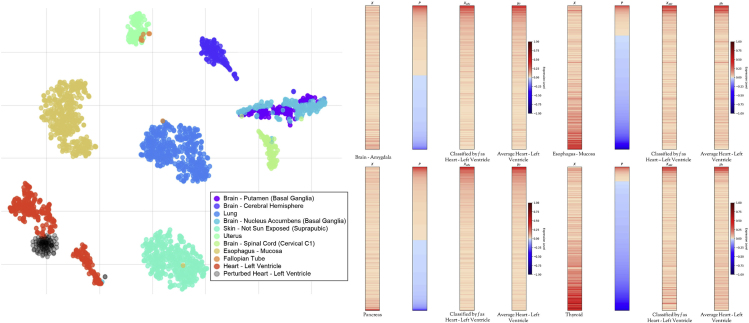
Adversarial Generation for Heart-Left Ventricle Target Using All Hallmark Genes as the Input Gene Set t-SNE plot of original and perturbed samples using the all Hallmark genes (left). Heatmap of cellular transformations from Brain-Amygdala, Esophagus-Mucosa, Pancreas, and Thyroid to Heart-Left Ventricle (right). Perturbations (*P*) range from [−1,1], which is added to original sample (*X*), then adversarial sample ($X_{\text{adv}}$) is clipped to [0,1]. The mean expression vector ($\mu_{T}$) of the target class (Heart-Left Ventricle) is shown.

Figure 2 shows the application of TSPG to a larger input gene set with all 4,386 Hallmark genes combined. In this case, the perturbed samples clustered with original samples of the target class (Heart-Left Ventricle). Additionally, the similarity between $x_{\text{adv}}$ and $\mu_{T}$ is apparent in the heatmaps. This experiment demonstrates the ability of TSPG to generalize to arbitrarily sized input gene sets. Figure S1 shows the results of the same experiment using Muscle-Skeletal condition as the target class, validating TSPG's ability to perturb toward varying conditions.

To clarify all applications of TSPG when perturbing toward varying conditions, we adopted a naming convention of Source→Target—Direction. For example, Whole-Blood→Nerve-Tibial—UP would be used to reference a perturbation from a Whole-Blood sample toward the class of Nerve-Tibial in the positive direction. The gene would be turned *up* in this case, indicating an activation of the gene during the transition toward Nerve-Tibial.

The classification accuracy, t-SNE visualizations, and perturbation heatmap results suggested that the perturbation of the Hallmark gene expression patterns successfully shifted the state of source tissue to target tissue. To test whether the perturbed genes were relevant to the source or target tissue, we randomly selected a sample from each of the 53 tissues available in the GTEx dataset and passed it through the generator that was trained with Heart-Left Ventricle as the target class. We then took the 20 most positively perturbed genes (Random→Heart-Left-Ventricle—UP) and the 20 most negatively perturbed genes (Random→Heart-Left-Ventricle—DOWN) for each of the 53 state transitions. Interestingly, a total of only 85 unique genes were identified in the top 20 across all 53 GTEx experiments out of the total 4,386 genes being positively perturbed. In other words, a distinct group of 85 genes were consistently perturbed in the positive direction moving toward the target tissue. This suggests that the same genes tend to be perturbed toward the target tissue, irrespective of the source tissue. On the other hand, the 20 most negatively perturbed genes across all target tissues combined to form 642 unique genes, suggesting that a greater variety of genes must be “turned off” depending on the phenotype of the source tissue.

We then tested whether the 20 most positively perturbed genes out of the pool of 4,386 genes were enriched (Benjamini-Hochberg false discovery rate [B&H FDR] q<0.0001) for biological function relevant to the target tissue (i.e., Heart-Left Ventricle). A representative enriched term for each of the 16 term categories and the total number of enriched terms across all 53 experiments is shown in Table 2. As observed, many of the enriched terms are related to muscle and heart biology. Conversely, we tested whether the 20 most negatively perturbed genes were related to the source tissue's biology. Table S1 shows the most significant PubMed scientific articles that were non-randomly associated with these genes. We found that the source tissue is related to the PubMed articles associated with these genes in most cases. All term enrichments for perturbations toward a Heart-Left Ventricle target tissue can be explored in Table S2.

**Table 2:** Enriched Biological Functions of the Top 20 Most Positively Perturbed Genes Leading to the Target Condition Heart-Left Ventricle

Term Category	Enriched Terms	First Hit Term Description	*q* Value
Coexpression	933	rat breast_Giusti09_300genes	1.72 × 10^−25^
Coexpression	985	heart muscle	8.01 × 10^−22^
Computational	315	neighborhood of MYL2	5.29 × 10^−22^
Disease	969	cardiomyopathy, familial idiopathic	1.47 × 10^−9^
Drug	1522	doxorubicin	2.88 × 10^−11^
Gene family	22	F-type ATPases|mitochondrial complex V	2.28 × 10^−11^
GO: BP	3311	striated muscle contraction	2.01 × 10^−15^
GO: CC	852	sarcomere	2.53 × 10^−15^
GO: MF	225	structural constituent of muscle	8.08 × 10^−6^
Phenotype	867	sudden death (human)	2.04 × 10^−7^
Interaction	280	VDAC1 interactions	1.94 × 10^−5^
MicroRNA	1	Hsa-miR-610:mirSVR	3.77 × 10^−5^
Phenotype	1419	cardiac hypertrophy (mouse)	2.47 × 10^−9^
Pathway	1340	cardiac muscle contraction	4.01 × 10^−14^
PubMed	10,774	clinical features and outcome of hypertrophic cardiomyopathy associated with triple sarcomere protein gene mutations.	1.13 × 10^−19^
ToppCell Atlas	209	mouse multiple adult muscle	2.90 × 10^−18^
		subtype heart_cardiac muscle cell	
TFBS	21	V$MEF2_02	8.40 × 10^−6^

**Figure 3:**
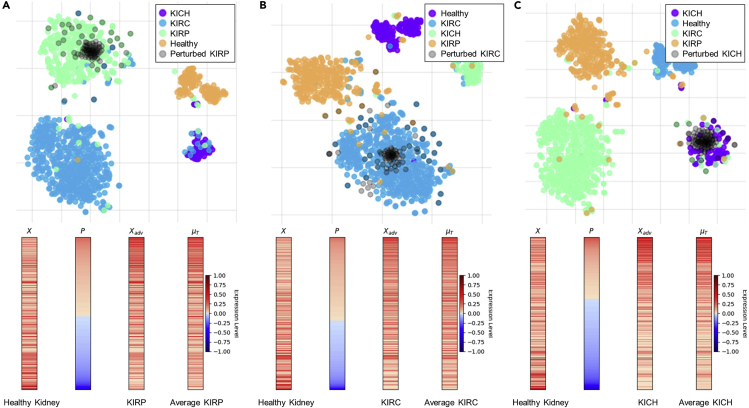
Adversarial Generation for Three Subtypes of Kidney Cancer Using All Hallmark Genes as the Input Gene Set t-SNE plot and corresponding heatmap of cellular transformation from healthy to KIRP (A), healthy to KIRC (B), and healthy to KICH (C). Perturbations (*P*) range from [−1,1], which is added to original sample (*X*), then adversarial sample ($X_{\text{adv}}$) is clipped to [0,1]. The mean expression vector ($\mu_{T}$) of the target class is shown.

To determine the efficacy of TSPG on more subtle transcriptome state transitions, we tested TSPG on samples from the same tissue of origin, namely healthy and cancerous kidney tissue extracted from the TCGA project. Using all 4,386 Hallmark genes as input, we examined the transcriptome transitions from healthy (“solid tissue normal”) into one of three renal tumor subtypes: clear cell renal carcinoma (KIRC), papillary renal cell carcinoma (KIRP), and chromophobe renal cell carcinoma (KICH) as shown in Figure 3. For each transition between healthy and the three tumor subtypes, we identified the genes that were among the top ten genes most positively and negatively perturbed during transition and performed functional enrichment to determine biological function of those genes (Table S3). For each case of the most negatively perturbed genes (Healthy-Kidney→KIRC/KIRP/KICH—DOWN), the genes tended to be enriched for top expressed genes in kidney tissue as defined by the GenitoUrinary Development Molecular Anatomy Project database.^39^ In contrast to the negatively perturbed genes appearing to have a normal kidney gene expression pattern, the top positively perturbed genes (Healthy-Kidney→KIRC/KIRP/KICH—UP) showed different enriched functions for each tumor subtype. A notable enriched function in KIRC genes was an association with genes involved in hypoxia, a hallmark of tumor progression whereby tumors avoid senescence by increasing blood supply (e.g., MSigDB hallmark hypoxia gene set was enriched). A notable function enriched in positively perturbed KIRP genes was an enrichment in membrane-binding annexin activity (e.g., Rescher and Gerke^40^), a dysregulated function in many cancers^41^ including renal carcinoma.^42^^,^^30^ Finally, positively perturbed genes from healthy to KICH exhibited an enrichment for genes upregulated by the estrogen receptor α (e.g., Stein et al.^43^), and estrogen-responsive genes have been implicated in kidney cancer.^44^ These results suggest that negatively perturbed genes exhibit similar functions when transitioning to any renal cell carcinoma (RCC) subtype, but the positively perturbed genes encode collective function unique to each subtype. Simply put, negatively perturbed genes are related to source tissue (i.e., healthy kidney) functionality, while positively perturbed genes relate to functionality specific to the target tissue (i.e., three cancerous kidney subtypes).

### Applying TSPG to Precision Medicine

**Figure 4:**
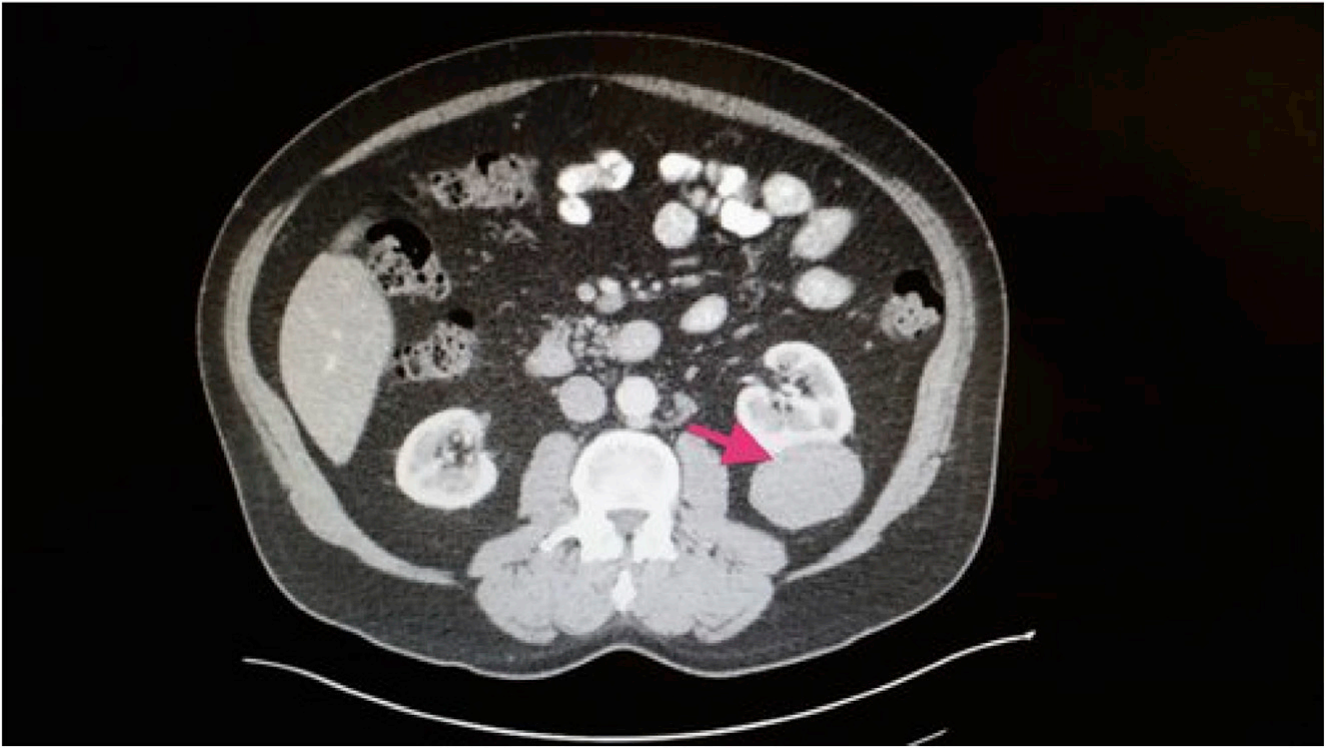
Computed Tomography Image of Patient BP's Renal Cell Carcinoma Imaged in February of 2014, this thoracic computed tomogram with intravenous contrast shows a 5.7 × 4.8-cm mass in the left kidney.

While these findings confirmed the efficacy of TSPG, we wanted to make this tool relevant for individual patients. The goal became to apply this deep-learning model to perturb a single sample of patient tumor RNA-seq data toward the normal phenotype of corresponding tissue, thereby determining patient-specific genomic aberrations from a single data sample. A patient (designated BP) was diagnosed with RCC in 2014 when a large mass was discovered in his left kidney (Figure 4). A month following the initial diagnosis BP underwent a total left nephrectomy, or surgical removal of the left kidney. Further histological analysis indicated that the cancer was stage 3 type I papillary RCC (KIRP, or p1RCC) and that it had not metastasized to the lymph nodes or other organs; however, it had infiltrated the renal fascia. Following this official diagnosis, facing a high risk of recurrence, BP obtained the RNA-seq data for his cancer as well as a sample of healthy resected kidney.^45^

BP's RNA-seq data was processed using the same method as for the TCGA and GTEx samples. The two processed vectors of BP's tumor RNA-seq sample and healthy tissue were then appended to a larger gene expression matrix (GEM) that included TCGA samples for KIRC, KIRP, and KICH, as well as the samples for healthy “solid tissue normal” TCGA and GTEx samples. The result was a normalized, comprehensive GEM consisting of three unique kidney cancer subtypes, healthy tissue data derived from two different sources, and patient-specific tumor/normal samples. An overview of the samples included in this comprehensive GEM is listed in Table 3.

**Table 3:** Sample Counts Included in the Comprehensive Kidney Cancer GEM

Tissue Type	Dataset of Origin	Count
KIRC tumor	TCGA	475
KIRC normal	TCGA	72
KIRP tumor	TCGA	236
KIRP normal	TCGA	29
KICH tumor	TCGA	60
KICH normal	TCGA	25
Kidney normal	GTEx	32
BP tumor	–	1
BP normal	–	1

**Figure 5:**
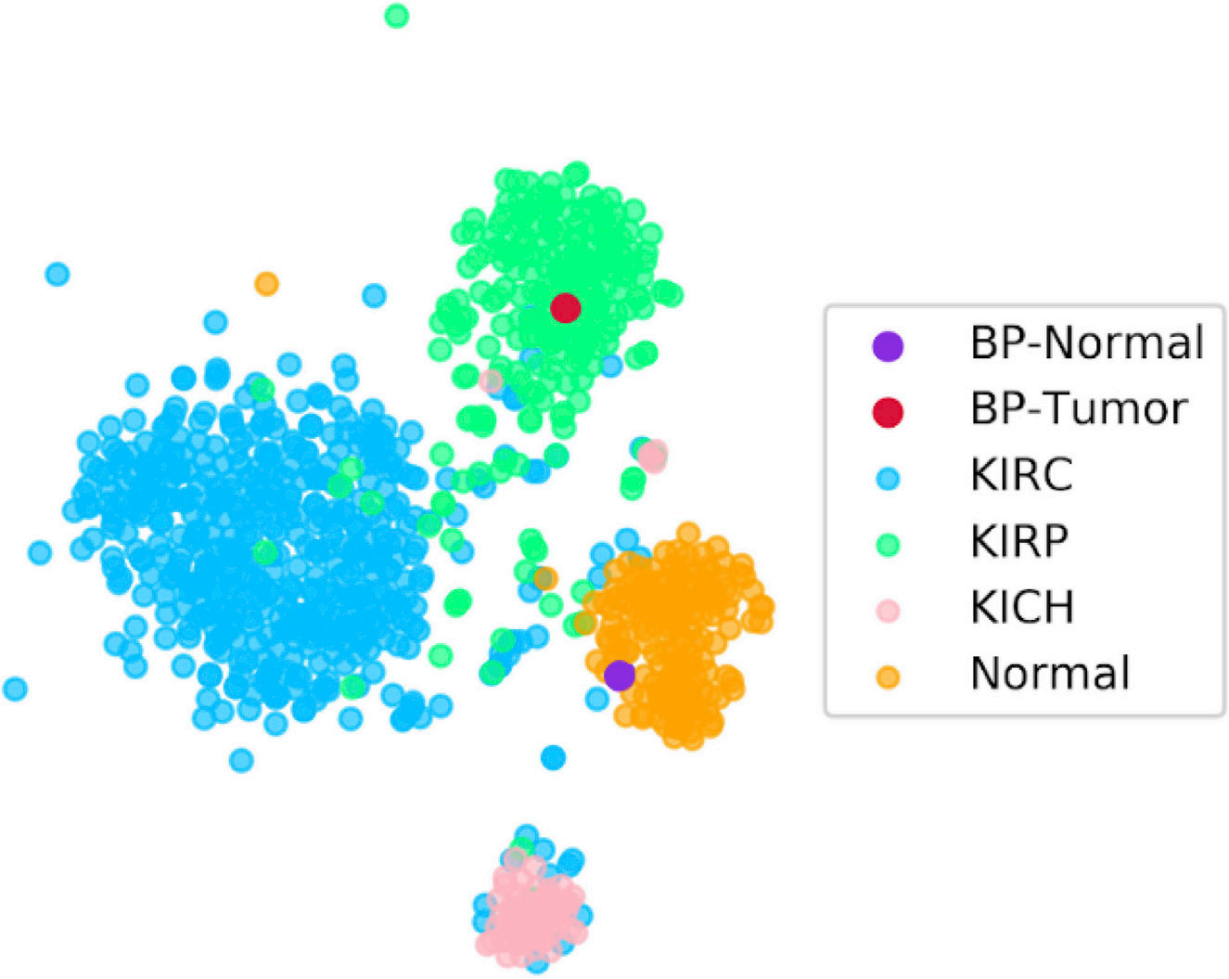
t-SNE Plot of Combined TCGA, GTEx, and Patient Data t-SNE visualization shows proper grouping between all combined normal samples and different RCC subtypes, with BP's sample clustering with the respective tissue group.

To ensure that this comprehensive GEM had been properly preprocessed so that BP's samples clustered with their histologically defined tissue subtypes, we again used t-SNE visualization to confirm that the unique vectors of expression data grouped with their respective tissue groups. GTEx was grouped with all other TCGA normal samples and given the common label of “Normal,” and was then compared against the other tumor subtypes. Figure 5 shows BP's normal sample clustering with the combined normal samples from GTEx and the different TCGA groups, as well as his tumor sample clustering with the relevant KIRP subtype.

**Figure 6:**
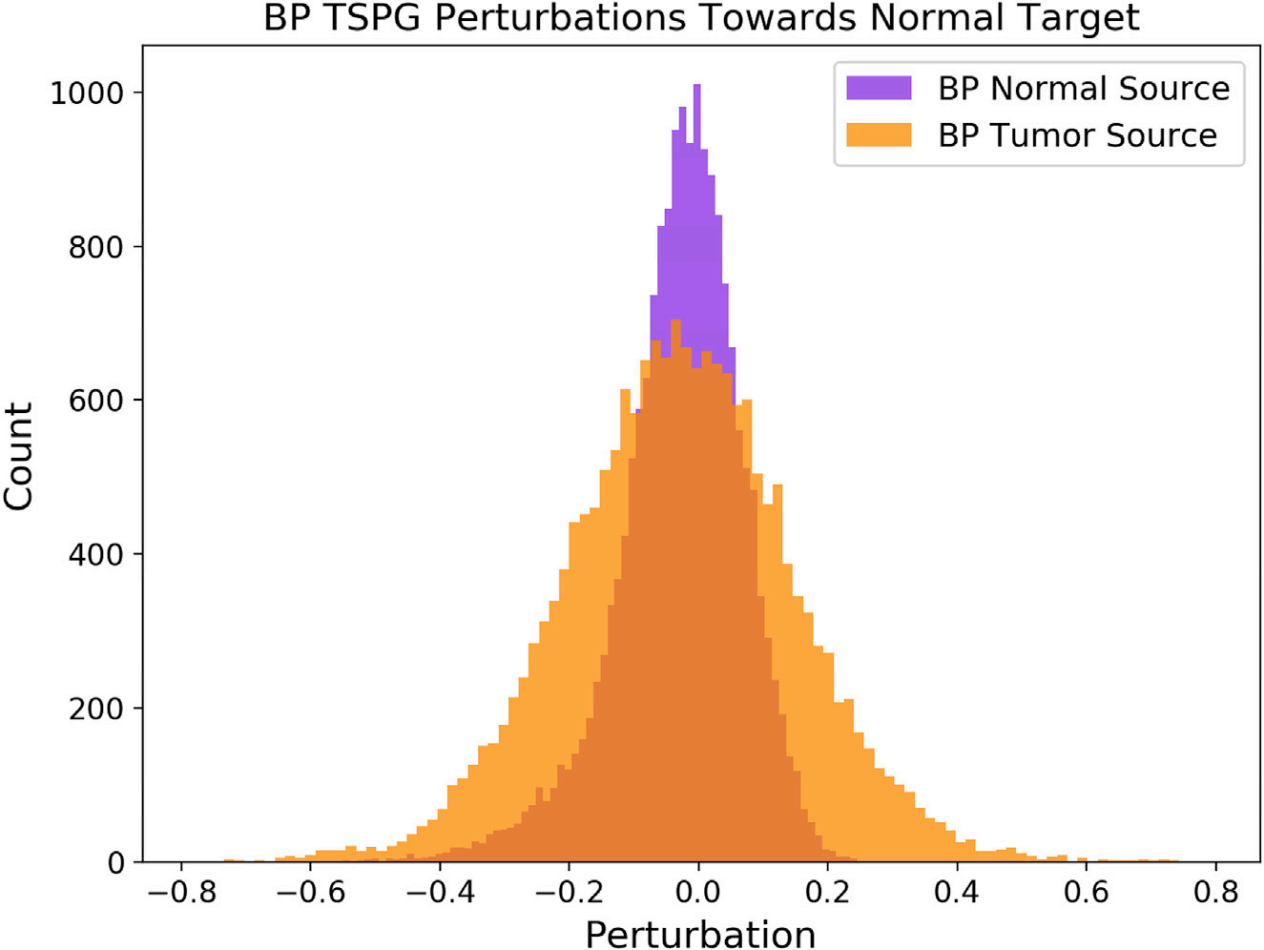
Distribution of TSPG Perturbations toward the Normal Target Class TSPG generates perturbations toward normal target tissue from a source of both BP's KIRP sample and sample of healthy kidney tissue. Perturbations from BP's normal sample toward the normal class show few significant transcriptional changes when compared with the perturbations from BP's tumor sample.

Following validation of the alignment of the comprehensive GEM, we used TSPG to perturb the expression values of BP's tumor sample toward a normal target class. Defining our perturbations in this direction allowed us to determine tumor-specific transcriptional aberrations from a single sample (i.e., n=1) of patient data once the TSPG model had been trained. BP's normal sample was perturbed toward the target class of normal as well, providing a naive comparison that displayed a narrower distribution of less extreme perturbations when compared with the perturbations from a tumor source (Figure 6). This histogram shows that when given the task to perturb a normal sample toward the normal class, the model makes significantly fewer changes than when perturbing from a source to a target class. In other words, the TSPG model does not generate significant perturbations unnecessarily. Furthermore, this histogram shows that the majority of genes in the tumor to normal transition remain unperturbed, indicating that much of the essential structure of the source tissue is maintained. Rather than nonsensically altering all genes to trick the classifier, the model preserves a great deal of transcriptional variation that is irrelevant to the difference between source and target.

To isolate the significant changes to the transcriptome state of BP's tumor compared with otherwise healthy kidney tissue, we isolated the perturbations determined by TSPG that lay more than two standard deviations outside of the mean. These genes, which had been significantly up- or downregulated in the change from tumor back to normal transcriptome states, were then divided by the direction of change. Positive perturbations reflected changes in those genes that needed to be upregulated to make the transcriptional state transition from tumor toward healthy kidney tissue (Tumor→Normal—UP). It is important to note that the experimental design for these patient-specific perturbations is reversed from the original kidney experiments reflected in Figure 3, as the purpose is no longer to test the efficacy of TSPG but rather to apply TSPG to an individual tumor sample, which means perturbing the tumor sample to appear normal. Tumor→Normal—UP perturbations, therefore, can be considered equivalent to Normal→Tumor—DOWN. Given the focus of these experiments was to consider tumor heterogeneity, we will therefore refer to all positive perturbations as tumor-downregulated genes. Simply put, tumor-downregulated genes are those which are expressed in lower levels in tumors relative to normal. Conversely, negative perturbations reflect changes in the genes that were most significantly downregulated when perturbing toward the class of normal kidney tissue (Tumor→Normal—DOWN). These genes will henceforth be referred to as tumor-upregulated genes, as these can be considered the genes more highly expressed in tumors relative to normal. TSPG identified 444 significant tumor-upregulated genes and 418 tumor-downregulated genes for BP's sample out of the total 18,368 included in the GEM.

Two particular genes of note, MET and ERRFI1, are of known relevance to RCC.^46^ Of the patients evaluated, four showed expression of the MET gene to be perturbed greater than one standard deviation from the mean degree of perturbation in the tumor-upregulated direction. Furthermore, five exhibited perturbations greater than one standard deviation in the tumor-downregulated direction for the ERRFI1 gene.

Functional enrichment was then performed on these two gene lists to search for biological functions relevant to the most highly perturbed genes in each direction (B&H FDR q<0.0001). Enrichment results for the tumor-downregulated list can be seen in Table 4, and results for the tumor-upregulated list are presented in Table 5. By performing functional enrichment for each perturbation direction independently, biological significance for source and target tissue can be separated. The 418 genes that were significant in the tumor-downregulated direction are involved in biological functions indicative of normal kidney function, while the 444 significant genes being perturbed in the tumor-upregulated direction indicate abnormal functions that may be representative of tumor-related functions. All significant enrichment results for both the tumor-upregulated and tumor-downregulated direction can be explored in Table S4.

**Table 4:** Enriched Biological Functions of the 418 Significant Tumor-Downregulated Genes Identified from BP's Tumor Sample

Term Category	Enriched Terms	First Hit Term Description	*q* Value
GO: MF	25	monovalent inorganic cation transmembrane transporter activity	1.832 × 10^−8^
GO: BP	78	ion transport	3.206 × 10^−12^
GO: CC	7	intrinsic component of plasma membrane	1.572 × 10^−14^
Human phenotype	7	abnormal circulating renin	8.091 × 10^−6^
Mouse phenotype	24	abnormal urination	2.289 × 10^−9^
Pathway	2	ensemble of genes encoding extracellular matrix and associated proteins	3.552 × 10^−6^
PubMed	56	activation of hypoxia signaling in stromal progenitors impairs kidney development^a^	3.805 × 10^−15^
Coexpression atlas	398	Kidney	5.459 × 10^−40^
TopCell atlas	83	epithelial—ts14 kidney of epithelial	7.531 × 10^−24^
Computational	3	heart, liver, kidney, and pancreas metabolic and xenobiotic response gene.	4.467 × 10^−7^
Disease	4	nephrocalcinosis	4.656 × 10^−6^

a Second ToppFun enrichment hit, noted because the first hit was a PubMed article relevant to mouse phenotype rather than human.

**Table 5:** Enriched Biological Functions of the 444 Significant Tumor-Upregulated Genes Identified from BP's Tumor Sample

Term Category	Enriched Terms	First Hit Term Description	*q* Value
GO: MF	8	G-protein-coupled receptor activity	2.935 × 10^−11^
GO: BP	22	keratinocyte differentiation	1.732 × 10^−15^
GO: CC	5	cornified envelope	3.827 × 10^−10^
Pathway	8	keratinization	5.231 × 10^−17^
PubMed	3	human olfactory receptor gene family	4.458 × 10^−11^
Gene family	2	keratins, type II	2.282 × 10^−5^
Coexpression	2	esophagus	7.487 × 10^−6^

### Comparison of TSPG Results between Patients

To examine the generalization of TSPG to multiple different vectors of similar data, we repeated this process with five other unique patient samples. Other patients were obtained by isolating TCGA samples that had correlating tumor/normal samples. The five other patients were chosen at random from the KIRP dataset. For each patient a unique comprehensive GEM was created by removing their normal and tumor samples to ensure mutual exclusivity between training and testing data. Creating a unique GEM for every patient's TSPG experiment maximized the amount of available training data for the model by including every tumor and normal sample that was not the patient's own while training the network. While generating unique input data for each experiment ensured optimal performance of the model, this posed a computational bottleneck resulting in our selective analysis of five TCGA patients. Despite selective analysis of only five patients, the design of TSPG leveraged the transcriptome data of 157 normal samples and 235 KIRP samples for each uniquely trained model while preserving one sample of each class for testing. These five experiments are highly parallel in nature and are therefore not to be understood as an attempt at a global analysis but rather as five replicates to demonstrate TSPG's applicability to many patients. Metadata was pulled from the Genomic Data Commons Data Portal, which houses the TCGA dataset,^47^^,^^45^ to convey patient demographic information, listed in Table 6. The complete TSPG results for all five TCGA patients, as well as those for BP, are available in Table S5.

**Table 6:** Patient Table for Each of the Randomly Chosen TCGA Data Points and for Patient BP

Sample ID	Age at Diagnosis	Race	Sex	Stage
TCGA-BQ-5884	59	AA	female	I
TCGA-BQ-7051	74	white	male	II
TCGA-DZ-6131	31	white	male	III
TCGA-GL-7966	28	white	female	III
TCGA-Y8-A8RY	63	white	male	I
BP	59	white	male	III

AA, African American.

**Figure 7:**
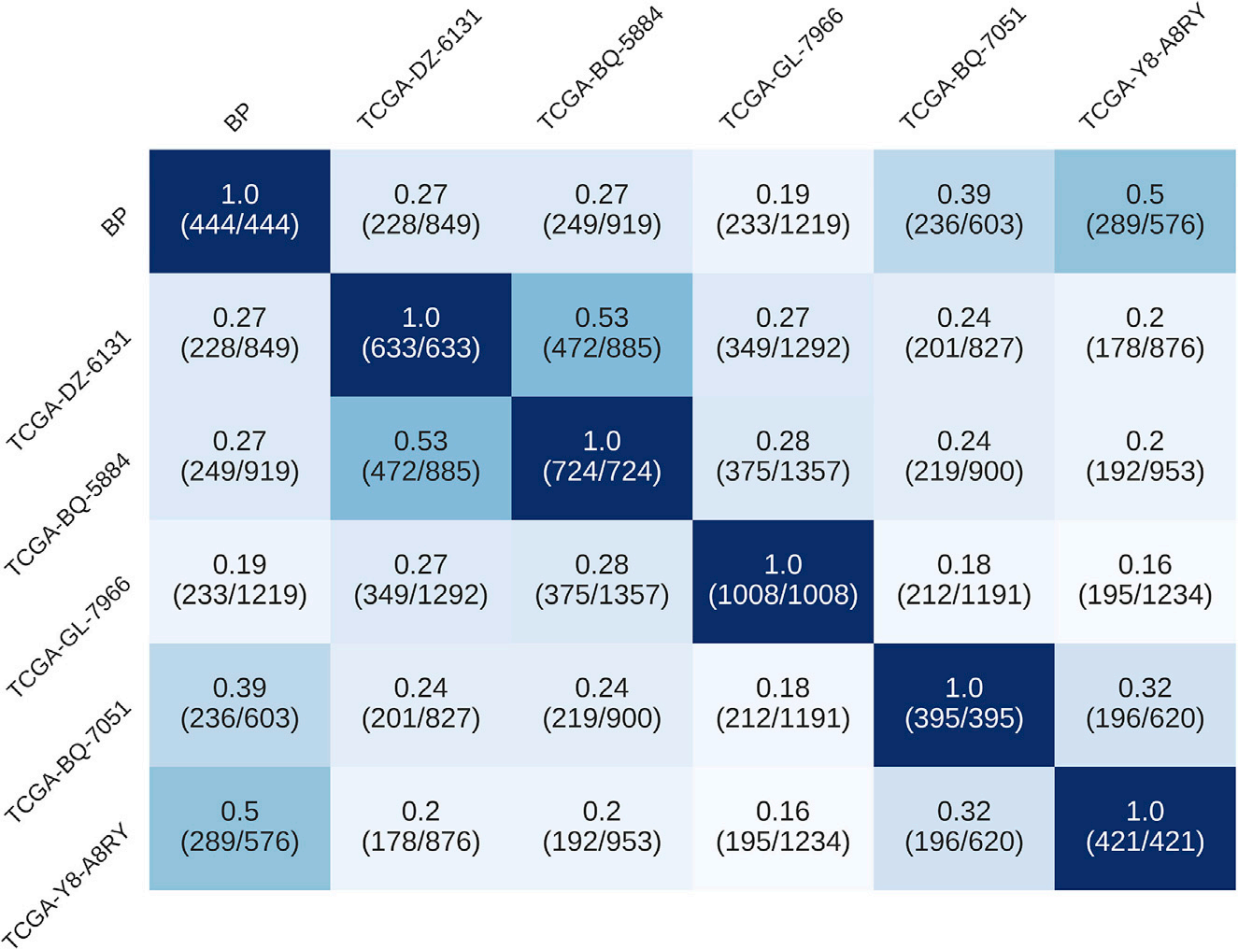
Heatmap Comparing the Overlapping Genes Identified by TSPG between Patients in the Tumor-Upregulated Direction Ratios are calculated as shared number of perturbed genes between two patients divided by the total number of unique perturbed genes found in both patients (numbers in parentheses).

**Figure 8:**
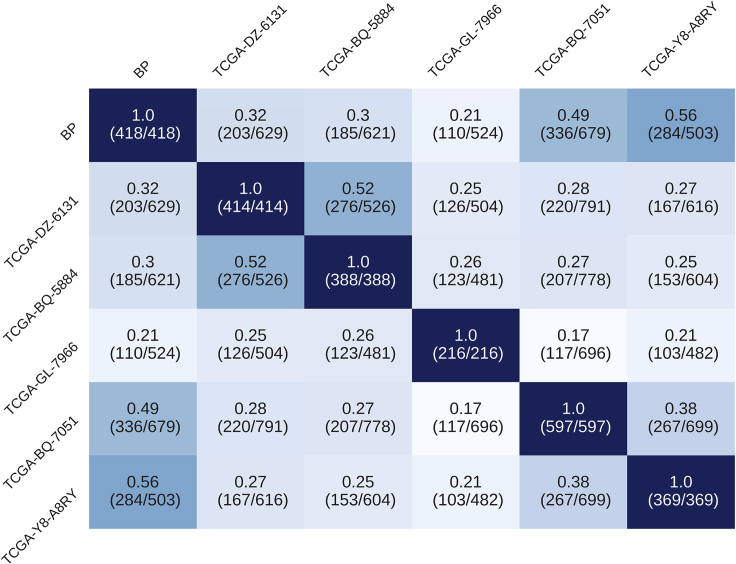
Heatmap Comparing the Overlapping Genes Identified by TSPG between Patients in the Tumor-Downregulated Direction Ratios are calculated as shared number of perturbed genes between two patients divided by the total number of unique perturbed genes found in both patients (numbers in parentheses).

All six TSPG results were compared against each other to determine overlap of the different perturbations between patients. For both the tumor-upregulated and tumor-downregulated directions, corresponding genomic perturbations were visualized to a heatmap in Figures 7 and 8. The comparison was performed by isolating the list of significant genes TSPG identified for patient *a*, appending the results from patient *b*, then taking the unique set of genes identified. The ratio then became (duplicate genes)/(all unique genes). Interestingly, although the general ratio of overlapping genes to total unique genes was similar between tumor-upregulated and tumor-downregulated genes, TSPG consistently isolated a wider variation between patients in different tumor-upregulated genes than it did tumor-downregulated genes, resulting in a larger set of total genes for the tumor-upregulated direction. The exception to this case is TCGA sample TCGA-BQ-7051, which can be explained by the fact that this is the only case in which TSPG generated more perturbations in the tumor-upregulated direction than in the tumor-downregulated direction (see Table 7). There is an evident pattern of TSPG identifying more unique tumor-upregulated genes than tumor-downregulated genes between patients. Statistical analysis by paired t test showed that TSPG identified significantly (p<0.0001) more unique tumor-upregulated genes between different patients (x=953) than tumor-downregulated genes (x=608), as is visualized in Figure S2. Genes upregulated in tumors relative to healthy kidney tissue are more unique between patients, being relevant to highly individualized cancer function, than those that are regularly downregulated across many patients.

**Table 7:** Percentage of Genes Identified by TSPG that Also Were Deemed Statistically Significant by DGE and Ratio between Overlapping Genes and All Significant Genes Identified by TSPG

Sample ID	Tumor-Upregulated	Tumor-Downregulated
TCGA-BQ-5884	21.4% (155/724)	64.1% (248/387)
TCGA-BQ-7051	32.9% (130/395)	78.6% (469/597)
TCGA-DZ-6131	26.9% (170/633)	70.7% (292/413)
TCGA-GL-7966	13.5% (136/1008)	75.5% (163/216)
TCGA-Y8-A8RY	39.4% (166/421)	82.4% (304/369)
BP	34.3% (152/444)	85.2% (356/418)

### Comparing TSPG with Differential Gene Expression

To further validate the perturbations generated by TSPG, we performed differential gene expression (DGE) analysis on the TCGA KIRP dataset compared with the GTEx normal kidney data and TCGA normal correlating tissue. DESeq2 was used perform the DGE analysis, which uses the negative binomial probability distribution to determine a reference by which to compare differential expression between classes.^48^ This type of statistical analysis is the standard for determining genetic differential expression between classes (e.g., tumor versus normal); however, it requires a large number of samples to achieve this binomial reference distribution. We therefore aimed to demonstrate that TSPG can achieve similar results by using a single sample on a pretrained model. The same data as listed in Table 3 was used, resulting in 236 KIRP samples being compared against 61 normal kidney samples for differential expression. A total of 6,863 genes were determined to be differentially expressed between KIRP and normal ($p_{\text{adj}}<0.0001$). Of those, 4,001 had a positive log_2_ fold change when comparing KIRP expression levels with a normal reference. These genes, being more transcriptionally active in a statistical majority of KIRP samples than in normal, can be correlated with those genes classified as tumor-upregulated from TSPG patient analyses. Alternatively, 2,862 of these genes had a negative log_2_ fold change identified by DGE. These genes can be correlated with those genes which TSPG perturbed in the tumor-downregulated direction, as these are the genes more highly expressed in the normal kidney tissue than in KIRP samples. Complete DGE results are listed in Table S6.

We then compared the genes that TSPG had most significantly perturbed from all five TCGA patients, as well as BP's, KIRP expression vectors with those DGE identified to be statistically significant to determine the overlap between the two different algorithm’ sets of results as depicted by Table 7. Notably, a large percentage of genes in the tumor-downregulated direction as determined by TSPG were also identified as statistically significant by DGE. Interestingly, however, a significantly lower percentage of genes identified by TSPG in the tumor-upregulated direction for each patient were deemed significant in the correlating direction by DGE.

The high correlation between the established method of DGE and the novel deep-learning model of TSPG in the tumor-downregulated direction further supports the efficacy of TSPG as a proposed alternative method of determining differential expression when given only one sample. Furthermore, it implies that the genes which TSPG independently identifies as being downregulated in tumors relative to normal tissue (tumor-downregulated) for each individual patient's tumor are in large part the same genes that are downregulated for the statistical majority of KIRP tumors as identified by DGE. In stark contrast, there is significantly less agreement between the genes that TSPG perturbed in the tumor-upregulated direction for each patient and those genes identified by DGE in the correlating direction using the binomial reference distribution. When considered in conjunction with the results from comparing the TSPG results from one patient to another, this further supports the conclusion that the genes perturbed in the tumor-upregulated direction are highly unique to each patient's tumor. While the genes perturbed in the tumor-downregulated direction largely match statistical averages of other KIRP tumors, the genes perturbed in the tumor-upregulated direction exhibit inherent heterogeneity both when compared directly with other tumors and when compared with statistical averages of all related tumors.

## Discussion

### Efficacy of TSPG

We adapted and added criteria to the AdvGAN framework, which originated from within the computer vision community, to produce meaningful biological state transitions. While we used $L_{2}$ regularization (Equation 3) to limit the magnitude of the perturbation, we observed that adding $L_{\text{td}}$ (Equation 4) to the generator loss function significantly improved the biological plausibility of the results. Furthermore, our experiments confirmed that neural networks used for classification of RNA-seq data are indeed vulnerable to adversarial “attacks.” The results of Table 1 show that it is actually quite trivial for the generator to “trick” the target model into a specific classification no matter what the input is. This is likely due to the fact that the target model is relatively simple and was not trained using any defensive strategies against attacks.

It is noteworthy that Szegedy et al.^28^ demonstrated that the fundamental nature of adversarial samples does not differ across models trained on different subsets of data or models that had varying hyperparameters (e.g., number of layers, number of neurons in each layer, weight normalization schemes). Thus, we decided it was not necessary to test across many different neural network architectures. Additionally, an important condition for adversarial attacks in image processing is that the perturbation should be small in magnitude (Equation 3); that is, the perturbation to an input should not be noticeable to the human eye. While this criterion is essential for adversarial attacks in the computer vision domain, we are more interested in making *meaningful* transitions from source to target states, not in meaninglessly fooling a neural network. Therefore, despite using $L_{2}$ regularization to stabilize training, it was not imperative that the perturbations be small in magnitude, since a large shift in gene expression may be required to transition between disparate tissue expression states.

What do these results mean for the study of biological systems? While the results of this analysis pointed to the near perfect adversarial attack rates (Table 1) and clear correspondence of the perturbed gene expression patterns to the target condition (Figures 1, 2, and 3), we were initially concerned that we had nonsensically transformed biological states. However, the strong signal of biological function (Tables 2 and S2) and continued selection of the same positively perturbed genes leading to the target gene expression pattern suggest that the algorithm shifts the expression patterns in a biologically meaningful way. Thus, we propose TSPG as a novel method to detect genetic subsystems that are responsible for differentiating between both differing tissues and wild-type and aberrant tissue states.

Generative models, particularly GANs, have been sparingly used in the field of biology due to a multitude of factors, including the limited number of adequate datasets and the highly preprocessed nature of RNA-seq data as compared with images and audio. This work serves as a demonstration that GANs can be applied to RNA-seq data to uncover meaningful gene expression patterns between different human tissue samples. We believe that GANs will enable novel approaches in the study of molecular biological systems.

### TSPG as an Alternative to Differential Gene Expression Analysis

The transcriptional state of tissue-specific cells is highly dynamic in nature, and its overwhelming complexity has been a barrier toward bringing this sequencing method to clinical applications. Given a single sample of patient data, it is extremely difficult to distinguish noise from relevant genomic information. Furthermore, statistical significance cannot be determined from a single data point, making it difficult to prove the validity of many findings. Therefore, it is of critical importance to develop novel methods to analyze the wealth of information provided by a single sample of patient RNA-seq data and transform it into clinically applicable results.^49^

DGE analysis is a well established method of determining differentially expressed genetic patterns between biological samples on a gene-by-gene basis. TSPG is proposed as an alternative approach to DGE that can identify differentially expressed genes using the global expression patterns. Thus, TSPG is a parallel approach that is applicable to a single patient's input after the model has been trained.

### TSPG Quantitatively Defines Tumor Genomic Heterogeneity

Malignancy of a cancer cell is largely understood to be the result of sequential alterations in the genetic, epigenetic, and transcriptomic architecture of the cell. Even within cancer types and subtypes, the transcriptomic state is highly variable. It is this stochastic nature of cancer that makes the response to various treatments highly variable between different patients.^50^ Tools such as DGE analysis lack the ability to evaluate the unique transcriptional changes that occur during tumorigenesis, as the goal of these tools is to look at changes that are evident in statistical averages of a population. TSPG, however, can identify changes that occur within a single sample.

Table 7 shows the similarity between genes identified by TSPG as significantly perturbed in the tumor-downregulated direction and those identified by DGE in the same direction as being differentially expressed for all TCGA KIRP samples. The significant overlap observed in this tumor-downregulated direction is evidence that there is a distinct subset of particular genes that are directly relevant to healthy kidney function and are repeatedly turned off during the progression of cancers. The tumor-upregulated direction, however, shows significantly less overlap with the DGE results. The genes identified by TSPG for each patient as being upregulated in cancer are highly variable and do not correlate as tightly with those that DGE identified as being relevant for statistical averages of the population of KIRP tumors. This low overlap between tumor-upregulated genes and those identified by DGE as being differentially expressed in the same direction is validation of the idea of tumor heterogeneity between patients. We propose that these genes that TSPG identifies, yet do not correlate with DGE findings, are not computational anomalies but rather can be indicative of patient-specific cancer biomarkers. While the unique transcriptional changes observed in BP may not signify genetic trends across all KIRP tumors, these patient-specific perturbations can be considered as candidate genes for targeted therapies specific to BP's cancer. TSPG can be used as a tool to identify novel biomarkers that are indicative of any source tissue if the model can be trained with the wealth of transcriptomic data available through publicly available sources.

### Identifying Papillary RCC Type-Specific Alterations

The seminal paper by Linehan et al. on the genomics of papillary RCC^46^ identified *MET* alterations as being largely characteristic of p1RCC. According to their characterizations, 81% of the 75 p1RCCs studied contained some alteration in the expression of the *MET* gene, which encodes a tyrosine kinase receptor protein. To this point, it was observed that BP's tumor, along with all five of the other patient tumors evaluated, showed overexpression of this *MET* gene. TSPG results for all six patients identified perturbations in the tumor-upregulated direction, and four of those (BP's included) showed perturbations greater than one standard deviation from the mean degree of perturbation. While this is a notably lower threshold than was considered for the majority of this study, it is worth noting that while the perturbations were less extreme, TSPG consistently identified a gene of known relevance to p1RCC to be overexpressed in all tumors evaluated. This further validation is supportive of the fact that *MET* plays a critical role in the progression of p1RCC and that TSPG is capable of highlighting even subtle transcriptional changes.

Deletion of the chromosomal region including tumor suppressor *ERRFI1*, a negative regulator of *EGFR*, was also observed in 11.1% of papillary RCCs.^46^ Of the six patients analyzed with TSPG, five exhibited perturbations greater than one standard deviation in the tumor-downregulated direction for this *ERRFI1* gene. This would indicate that expression of the *ERRFI1* gene is significantly lower in papillary RCC when compared with healthy kidney tissue, consistent with a deletion or silencing of the gene. Although these are merely observational correlations, it is notable that some of the most critical genetic alterations of papillary RCC identified by Linehan et al.^46^ are independently identified by TSPG. Papillary RCC is an extremely heterogeneous disease characterized by many modes of disease progression and unique genetic drivers. While type I papillary RCC is the most commonly diagnosed, there remains a large array of different histologically unique subtypes. The larger collection of non-type-I papillary RCCs is composed of type II papillary renal carcinomas, including duct carcinomas, medullary renal cell carcinomas, the MiTF kidney cancers (TFE3, TFEB), and hereditary leiomyomatosis RCC-associated kidney cancers.^51^ All of these unique histologies exhibit unique genetic profiles; however, there is much to be learned about the differentiating genetic characteristics of each. Given that TCGA provides a common label to all papillary RCC tumors, it is difficult to profile transcriptional changes in a systematic method across each patient. As more becomes known about the distinctive markers of each, and increasingly specific labels can be provided to public data stores such as TCGA, it will be possible to use TSPG to characterize these tumors based on specific transcriptional aberrations. Future studies should analyze patients of known histology, as was the case with BP, to verify in more detail many of these transcriptional changes.

### Understanding the Transcriptional Aberrations of BP's p1RCC

Functional enrichment results for BP's 418 significantly perturbed tumor-downregulated genes show consistent enrichment for many functions that are relevant to kidney function and development. Enrichment results for functions such as urination, circulating renin, kidney coexpression, and a diseased state of nephrocalcinosis, as shown in Table 4, all indicate that TSPG is in fact identifying genes that support biologically relevant kidney function. Being that these are the genes being downregulated in the tumor, expression of these genes must be turned back up in order to reflect the transcriptional state of healthy kidney tissue. It makes sense, therefore, that these genes identified by TSPG as being extremely perturbed in the state transition from tumor to healthy kidney be enriched for kidney function.

To understand the transcriptional changes that occurred during progression of BP's p1RCC, we can analyze the 444 genes that TSPG identified as being significantly perturbed in the tumor-upregulated direction. Interestingly, consistent significant enrichment for keratinization function was observed (see Table 5). Keratins are intermediate filaments of the epithelial cytoskeleton, and tubular epithelial cells have been previously identified as highly differentiated renal cells essential for normal kidney function.^52^ Keratins are known to be upregulated in various disease states including skin disease and cancer as well as pancreatic and liver injury. Previous studies have identified keratins as markers of renal epithelial cell injury, being upregulated when renal cells are under stress.^53^ This indicates that the upregulated genes in BP's tumor are enriched for cellular function, which has been previously associated with kidney injury and cancer.

With this initial validation of the potential clinical relevance of the genes that TSPG can identify, future studies should aim to determine regulatory pathways that affect these genes, ultimately aiming to identify therapeutics that specifically target those pathways. Furthermore, future aims involve collaborating with wet lab biologists to perform immunohistochemistry and western blotting on BP's preserved tumor tissue sample to confirm that the identified candidate genes are in fact being overexpressed in the cells. This study provides a computational tool for analyzing gene expression data that can predict biologically significant genetic aberrations. Further research should focus on determining biological and clinical validity of these predictions to establish a bench-to-bedside workflow. This is the ultimate step in bringing TSPG to a clinically applicable setting where precision medicine can be applied based on this deeper understanding of transcriptional activity of cancer cells.

## Experimental Procedures

### Resource Availability

#### Lead Contact

F. Alex Feltus is the lead contact for this study.

#### Materials Availability

There are no physical materials associated with this study.

#### Data and Code Availability

a The source code for TSPG along with all documentation is available at the following Github repository: https://github.com/ctargon/TSPG
b The normalized gene expression data (FPKM) from TCGA and GTEx data are available on *Figshare*.^54^ https://doi.org/10.6084/m9.figshare.5330593
c The exact parameters used in the workflow to process patient-derived FASTQ data to be compared with public sources can be found at the following Github repository: https://github.com/mrbende/RNAprep
d Maximum likelihood gene expression counts from TCGA and GTEx data are available on *Figshare*.^54^ https://doi.org/10.6084/m9.figshare.5330539

### Input Data

We used two GEMs containing RNA-seq expression levels for human tissue and tumor samples: (1) The GTEx dataset (11,688 samples by 56,202 genes representing 53 uniquely labeled tissue types);^37^ (2) TCGA dataset (11,092 samples by 60,483 genes representing 33 unique tumor types).^55^ We performed log_2_ transformation and quantile normalization on the GEM in Python using scikit-learn.^56^ We then used the Broad Institute Molecular Signature Database gene subsets (MSigDB v6.2) (MSigDB)^36^ to extract normalized sub-GEMs from GTEx and TCGA. These sub-GEMs were normalized using a min-max scaler, which scales each feature to the range [0,1].

### TSPG Design

**Figure 9:**
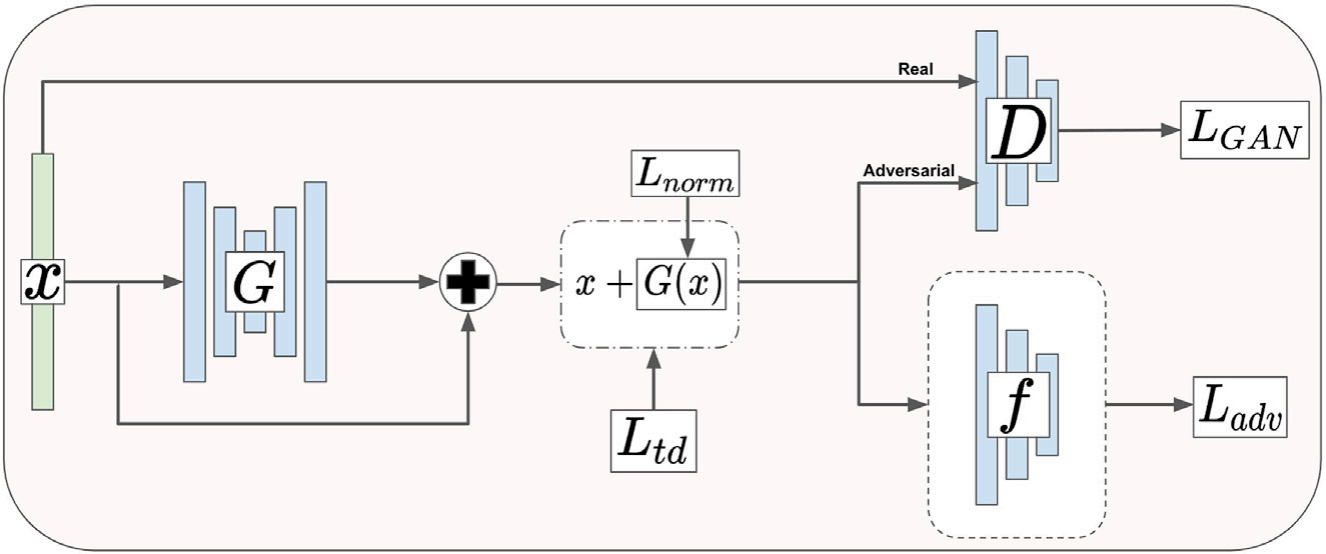
Architecture of Transcriptome State Perturbation Generator

The TSPG framework, shown in Figure 9, was adapted from the AdvGAN model introduced by Xiao et al.^32^ TSPG consists of a generator *G*, a discriminator *D*, and a target model *f*. The generator *G* takes an *n*-dimensional sample *x*, where *n* is the number of genes, and generates a perturbation G(x). G(x) is added to the original sample to produce an adversarial sample $x_{\text{adv}}=x+G\left(x\right)$ which is then passed to both the discriminator and the target model. The discriminator *D* outputs a prediction of whether the generated sample came from the training data or the generator (whether $x_{\text{adv}}$ is “real” or “fake”). The goal of the discriminator is to encourage the generated sample $x_{\text{adv}}$ to be indistinguishable from the training data, and this goal is quantified by the loss term,

(Equation 1) $$L_{\text{GAN}}=E_{x}\left[\text{log}D\left(x\right)\right]-E_{x}\left[\text{log}\left(1-D\left(x+G\left(x\right)\right)\right)\right]\text{.}$$

For $L_{\text{GAN}}$, we use the least-squares objective proposed in Mao et al.^57^ to stabilize training and boost results. The target model *f* outputs a class prediction for input $x_{\text{adv}}$ based on its pretrained weights. The goal of the target model is to learn a mapping from the training data to the training labels, and the goal of the generator is to produce an adversarial sample $x_{\text{adv}}$ such that the target model classifies $x_{\text{adv}}$ incorrectly as class *t*. The loss term for “tricking” the target model in this way, proposed by Carlini and Wagner,^30^, is

(Equation 2) $$L_{\text{adv}}^{f}=\text{max}\left({\text{max}}_{i\neq t}f{\left(x_{\text{adv}}\right)}_{i}-f{\left(x_{\text{adv}}\right)}_{t},\kappa \right)\text{,}$$

where *t* is the target class, and we set confidence κ=0. This loss term encourages the generator to produce data that fools *f* into predicting *t*.

We also use two terms, $L_{\text{norm}}$ and $L_{\text{td}}$, which are used to stabilize training and encourage $x_{\text{adv}}$ to fall within the target data distribution $P_{t}$. $L_{\text{norm}}$ is simply the $L_{2}$ norm placed on the generated perturbation G(x):

(Equation 3) $$L_{\text{norm}}={\left\|G\left(x\right)\right\|}_{2}\text{.}$$

This loss term encourages the overall perturbation to be small and also stabilizes training with better results. The final loss term, $L_{\text{td}}$, is the $L_{1}$ distance between an adversarial sample $x_{\text{adv}}$ and a randomly sampled target vector $r_{t}$, where $r_{t}∼N\left(\mu_{t},{\text{Σ}}_{t}\right)$. $N\left(\mu_{t},{\text{Σ}}_{t}\right)$ is a normal distribution modeled with the mean *μ* and covariance matrix Σ of target class *t*. This loss term is formulated as

(Equation 4) $$L_{\text{td}}=\left|x_{\text{adv}}-r_{t}\right|\text{.}$$

$L_{\text{td}}$ encourages the generator to create data more tightly within the bounds of the target class distribution. Similar principles are used in Isola et al. and Zhu et al.^25^^,^^58^ to reduce the space of possible mappings and create more realistic data samples. Together, the loss for *G* can be summed as

(Equation 5) $$L=L_{\text{GAN}}+L_{\text{adv}}+L_{\text{norm}}+L_{\text{td}}\text{.}$$

The architectures used by *G* and *D* are similar to those used by Xiao et al.,^32^ which draw influence from image-to-image translation architectures.^25^^,^^58^ *G* uses an hourglass-shaped structure which contains three fully connected layers of sizes 512, 256, and 128, followed by three residual blocks, followed by two layers of sizes 256 and 512 and then a layer of the same shape as the input dimension. Each dense layer is coupled with batch normalization and rectified linear unit (ReLU) activation, and the output layer uses the hyperbolic tangent (tanh). The tanh function allows the generator to produce a perturbation ranging from [−1,1]. This perturbation can be interpreted roughly as a normalized log_2_ fold change. The discriminator consists of three dense layers of sizes 512, 256, and 128. Each dense layer is followed by a batch normalization layer and a leaky ReLU activation function with alpha set to 0.2. The target model used in the experiments contains three dense layers of size 1,024, 512, and 128, each layer using the ReLU activation function.

All three networks were trained using the Adam optimizer^59^ with learning rates 0.0002, 0.0001, and 0.001 for the generator, discriminator, and target model, respectively. The target model used bootstrapping by randomly selecting 90% of each class for training and holding out the remaining 10% for testing, and it was trained for 30 epochs with a minibatch size of 32. Similarly, the generator used an 80%/20% split and was trained for 150 epochs with a minibatch size of 128. All three networks were developed in TensorFlow^60^ and trained on Clemson University's Palmetto Cluster using NVIDIA V100 GPUs. Source code and documentation for TSPG are available at Data and Code Record *a*.

### TSPG Workflow

The TSPG workflow consists of three steps:

#### Train the Target Model

The target model is trained on the training data, which in our case is the combined GTEx and TCGA GEM or a subset thereof. This is the model which assigns a class label to a sample of transcription data.

#### Train the Perturbation Generator

The generator is trained (using the same training data as the target model) to generate perturbations toward a given target class. The perturbed samples should be classified as the target class by the target model and should be indistinguishable from the training data according to the discriminator.

#### Generate Sample Perturbations

The generator is used to perturb each sample in the test data to “appear like” the target class. The perturbed test samples are considered valid if they are classified as the target class by the target model. These perturbations can then be visualized or used for other downstream analyses such as functional enrichment.

### Initial Validation

To validate TSPG, we trained the target model and the generator on the combined GTEx and TCGA data, then we perturbed 100 randomly selected samples from these training data along with the samples from 10 randomly selected classes including the target class. t-SNE plots were used to visualize both original and perturbed samples. Additionally, we used the mean vector of a target class to visually inspect how similar an adversarial sample is to the target class.

### Unifying Patient Data with TCGA/GTEx

The process for unifying TCGA tumor data with GTEx tissue data was documented by Wang et al.,^54^ and the data are freely available from Data and Code Record *b*. The following files were pulled for analysis:

1. kich-rsem-fpkm-tcga.txt
2. kich-rsem-fpkm-tcga-t.txt
3. kirc-rsem-fpkm-tcga.txt
4. kirc-rsem-fpkm-tcga-t.txt
5. kirp-rsem-fpkm-tcga.txt
6. kirp-rsem-fpkm-tcga-t.txt
7. kidney-rsem-fpkm-gtex.txt

By pulling only relevant kidney data, the size of the dataset was minimized while still providing several subtypes of kidney tissues for analysis to avoid overfitting the TSPG model. These datasets were then merged along gene IDs.

Illumina RNA-seq was performed on both a biopsy sample of BP's p1RCC and healthy kidney tissue, resulting in two single-insert paired-end RNA-seq datasets in FASTQ format. The normal sample was sequenced with 150M requested reads and the p1RCC sample was sequenced with 300M reads. These FASTQ samples were processed according to protocols used for processing this GTEx and TCGA data.^54^ Alignment was performed using STAR aligner v2.4.2a with the hg19 build of the University of California Santa Cruz human reference genome and the GENCODE comprehensive gene annotation list.^61^ Quantification of gene expression data was performed using RSEM.^62^ These two expression vectors (normal and tumor) were then appended to the larger GEM of GTEx and TCGA kidney data from Wang et al.^54^ and normalized in the same way as described previously, so that the patient data were accurately aligned with a larger collection of public data. To verify that BP's samples had been properly merged with the larger GEM while retaining realistic gene expression patterns, we used t-SNE visualization to validate proper clustering of tissue types. The result of this unification is a single GEM consisting of GTEx and TCGA data along with aligned patient-specific data. The exact parameters used when running these workflows is available at the repository listed in Data and Code Record *c*.

### Functional Enrichment Analysis

To check for biological relevance, we used the ToppFun tool (https://toppgene.cchmc.org)^63^ to perform functional enrichment analysis. Occasionally, the HGNC symbol was not recognized for 1/20 genes in the list. Functional labels were considered significant at q<0.0001.

### Running TSPG on Patient-Specific Data

To perform the patient-specific analysis, we trained the target model and the generator on the kidney data from GTEx and TCGA (i.e., excluding the patient's data). This training set contained 158 normal samples, 60 KICH samples, 475 KIRC samples, and 236 KIRP samples. The generator was trained to perturb the input to appear normal so that we could observe the transition from the patient's tumor sample to normal. After the target model and the generator were trained, we generated perturbations for both the patient-normal sample and the patient-tumor sample. A naive comparison was also performed by taking the difference between these two samples, which served as a baseline against which to compare the actual results of TSPG. By making these perturbations based on the 158 available normal samples rather than just the one patient-normal sample, TSPG was able to circumvent the high uncertainty associated with an n=1 analysis while retaining patient-specific information. All genes from the patient samples were used in this analysis to avoid the potential loss of relevant gene perturbations.

### Differential Gene Expression Analysis

DGE analysis was performed using the Bioconductor package DESeq2.^64^ Rather than using normalized estimated expression levels, raw counts of sequencing reads were used. These datasets were published by Wang et al.^54^ alongside the normalized expression values and are available for download from Data and Code Record *d*. DGE analysis was performed using the “kirp-rsem-count-tcga-t.txt” dataset as the source group, while “kirp-rsem-count-tcga.txt” and “kidney-rsem-count-gtex.txt” were used together as the target group. This was intended to replicate TSPG's function of measuring transitions between an aberrant source group toward a normal target group. t-SNE visualization was again used to confirm proper clustering of source and target groups. To analyze the results of DGE, we isolated statistical outliers that represented the most highly differentially expressed genes (both up- and downregulated) ($p_{\text{adj}}<0.0001$). These were then separated by the direction (positive value reflecting an upregulation in the shift from KIRP to normal, negative reflecting downregulation) of the log_2_ fold change.

## Supplemental Information

Document S1. Figures S1 and S2

Table S1. Most Significant PubMed Articles Associated with the Top 20 Most Negatively Perturbed Genes from Source Tissue

Table S2. Enriched Gene Function Analysis of Top 20 Most Perturbed (POS) or Least Perturbed (NEG) Hallmark Genes from the Source Tissue to the Target Tissue

Table S3. Enriched Gene Function Analysis for Kidney Tissue of Top Ten Most Perturbed (POS) or Least Perturbed (NEG) Hallmark Genes

Table S4. Functional Enrichment Results for the Tumor-Upregulated genes (n=444) and Tumor-Downregulated genes (n=418) which TSPG Identified as Significant for BP’s Tumor Sample Perturbations Toward Normal

Table S5. TSPG Perturbations for All Five TCGA Patients and for BP toward a Normal Target Tissue

Table S6. Differential Gene Expression Analysis Comparing KIRP with Normal Samples

Document S2. Article plus Supplemental Information
