# Supplementary material for: Cellular State Transformations Using Deep Learning for Precision Medicine Applications
Source: Patterns (N Y). 2020 Aug 17;1(6):100087. doi: 10.1016/j.patter.2020.100087 (PMC7660411; doi:10.1016/j.patter.2020.100087)
Supplement: Document S1. Figures S1 and S2 [file mmc1.pdf]

PATTER, Volume *1*

Supplemental Information

Cellular State Transformations Using Deep Learning for Precision Medicine Applications

Colin Targonski, M. Reed Bender, Benjamin T. Shealy, Benafsh Husain, Bill Paseman, Melissa C. Smith, and F. Alex Feltus

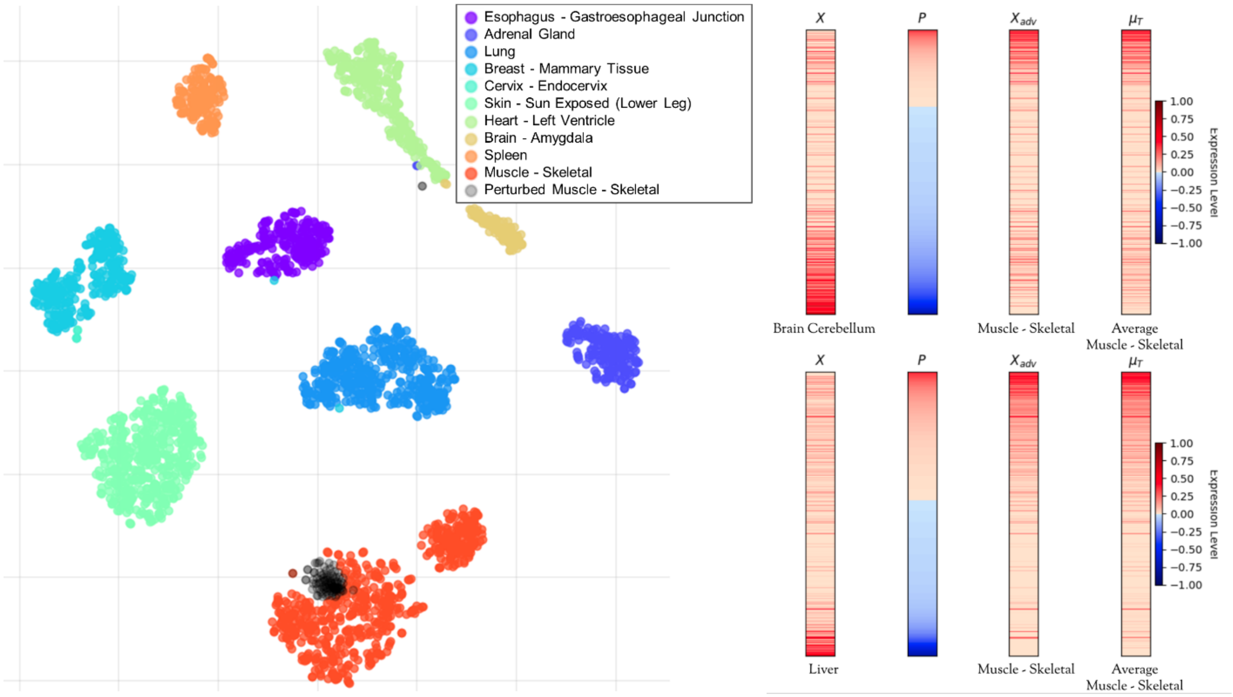

Supplemental Figure S1. Adversarial Generation for Muscle-Skeletal Target Using all Hallmark Genes as the Input Gene Set. t-SNE plot of original and perturbed samples using the all Hallmark genes (left). Heatmap of cellular transformations from Brain-Cerebellum and Liver to Muscle-Skeletal (right). Perturbations (*P*) range from [-1,1], which is added to original sample (*x*), then adversarial sample (x_{adv}) is clipped to [0,1]. The mean expression vector (μ_T) of the target class (Muscle-Skeletal) is shown.

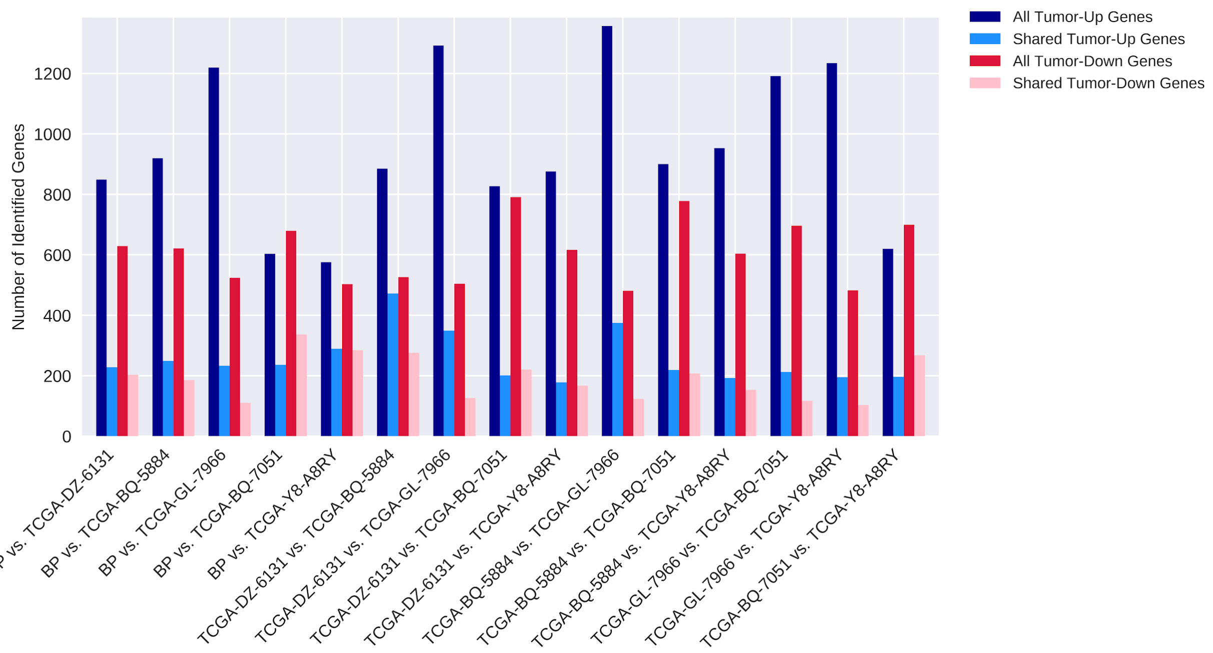

Supplemental Figure S2. Uniquely Perturbed Genes in Kidney Tumors. Comparison of TSPG results between patients shows a pattern of more unique *tumor-upregulated* genes being identified than *tumor-downregulated* genes.
